# Emerging Immune-Monitoring System for Immune Checkpoint Inhibitors

**DOI:** 10.3390/life12081229

**Published:** 2022-08-13

**Authors:** Kazuyuki Hamada, Takuya Tsunoda, Kiyoshi Yoshimura

**Affiliations:** 1 Division of Medical Oncology, Department of Medicine, Showa University School of Medicine, Tokyo 142-8555, Japan; 2 Department of Clinical Immuno-Oncology, Clinical Research Institute of Clinical Pharmacology and Therapeutics, Showa University, Tokyo 157-8577, Japan

**Keywords:** immune checkpoint inhibitors, intestinal bacteria, biomarkers, PD-L1 expression, cancer microenvironment, gut microbiota

## Abstract

Immune checkpoint inhibitors (ICIs) have a major impact on cancer treatment. However, the therapeutic efficacy of ICIs is only effective in some patients. Programmed death ligand 1 (PD-L1), tumor mutation burden (TMB), and high-frequency microsatellite instability (MSI-high) are markers that predict the efficacy of ICIs but are not universally used in many carcinomas. The gut microbiota has received much attention recently because of its potential to have a significant impact on immune cells in the cancer microenvironment. Metabolites of the gut microbiota modulate immunity and have a strong influence on the therapeutic efficacy of ICI. It has been suggested that the gut microbiota may serve as a novel marker to predict the therapeutic efficacy of ICI. Therefore, there is an urgent need to develop biomarkers that can predict anti-tumor effects and adverse events, and the study of the gut microbiota is essential in this regard.

## 1. Introduction

Immune checkpoint inhibitors (ICIs) have a significant impact on cancer treatment. However, further improvement in the efficacy of ICIs is required. For this reason, there is an urgent need for biomarkers of high efficacy and prognostic value. Therefore, it is important to investigate the conditions of the cancer microenvironment in which the efficacy of ICIs can be demonstrated. The expression of programmed death ligand 1 (PD-L1) on tumor cells, tumor mutation burden (TMB), and high-frequency microsatellite instability (MSI-high) have been reported as biomarkers for the efficacy of anti-PD-1/PD-L1 antibodies. However, the expression of PD-L1 has limitations as a biomarker because of its heterogeneity within and between tumors. Additionally, PD-L1 expression itself is transient and changes with the immune response over time and space. In the IMpower 133 trial, the efficacy of carboplatin and etoposide with atezolizumab, an anti-PD-L1 antibody, was analyzed based on the PD-L1 expression on tumor cells, but there was no difference in the efficacy between patients with PD-L1 expression and those without [1]. There is no clear biomarker for anti-PD-L1 combination chemotherapy for small cell lung cancer [1]. In addition, intestinal bacteria have been attracting attention in recent years because they may have a significant impact on the immune cells in the cancer microenvironment.

In addition to monotherapy with anti-PD-1/PD-L1 antibodies, combination therapy with chemotherapy and molecular target therapy as well as combination therapy with anti-PD-1 and anti-cytotoxic T lymphocyte antigen-4 (anti-CTLA-4) antibodies is now widely used in cancer treatment with ICIs [2]. As a result, there is an urgent need to develop biomarkers that can predict the anti-tumor response and adverse events, and research on intestinal bacteria is essential in this regard.

## 2. Mechanism of Action of Anti-PD-1/PD-L1 and Anti-CTLA-4 Antibodies

The immunosuppressive immune checkpoints pathway is an important immune resistance mechanism in cancer [3]. Programmed cell death 1 (PD-1) is one of the critical immune checkpoint receptors that are primarily expressed on a variety of immune cells, including activated T, B, regulatory T (Treg), and dendritic cells (DC) [4]. PD-1 and PD-L1 are immune checkpoints that regulate adaptive immune responses to prevent excessive activation and maintain immune homeostasis [3,4]. Anti-PD-1/PD-L1 antibodies mainly suppress the negative regulatory mechanisms between tumors and T cells. This is referred to as the effector phase [5]. The anti-PD-1 antibodies pembrolizumab and nivolumab showed promising results in non-small cell lung cancer (NSCLC) and melanoma patients [6,7,8]. Subsequently, their efficacy was demonstrated in many cancers, including urothelial bladder cancer, renal cell carcinoma, gastric cancer, Hodgkin’s lymphoma, and head and neck squamous cell carcinoma [9,10,11,12,13,14]. Atezolizumab was the first PD-L1 antibody to be approved by the FDA for non-small cell lung cancer patients [15]. Avelumab showed efficacy against Merkel cell carcinoma [16].

Furthermore, atetzolizumab administered after adjuvant chemotherapy in patients with resected stage IB-IIIA non-small cell lung cancer statistically significantly prolonged disease-free survival compared to best supportive care after adjuvant chemotherapy [17].

In contrast, anti-CTLA-4 antibodies maintain T-cell activation by blocking inhibitory signals from dendritic cells in the lymph nodes [18]. This phase is called the priming phase [5]. The primary mechanism by which CTLA-4 suppresses T-cell activation is through transendocytosis, which traps the ligand CD86 from DCs and sends a negative signal to T cells. Transendocytosis can be prevented by the anti-CTLA-4 antibody, which maintains the expression of the CD86 ligand [19]. Transendocytosis of CD86 on Tregs results in the removal of the ligand from antigen-presenting cells (APCs) and prevents normal T-cell activation, resulting in an antitumor effect [20]. Ipilimumab, a CTLA-4 inhibitor, was the first approved ICI for patients with advanced melanoma [21,22] (Table 1).

## 3. PD-L1 Expression in Tumor Cells

PD-L1 is a ligand for PD-1 [4]. PD-L1 has been used as a biomarker to predict clinical response to PD-1 or PD-L1 antibody therapy [26]. From 2011 through April 2019, only some immune checkpoint inhibitors approved by the US Food and Drug Administration (FDA) approved PD-L1 as a companion diagnostic [27]. During the above time period, FDA-approved immune checkpoint inhibitor carcinomas included non-small cell lung cancer (NSCLC), melanoma, bladder, renal, head and neck, colon, hepatocellular, small cell lung cancer, Merkel cell carcinoma, squamous cell carcinoma of the skin, Hodgkin lymphoma, breast, gastric/gastroesophageal junction (GEJ), primary mediastinal B-cell lymphoma, and MSI-high/mismatch repair-deficient status [27]. Among the above, PD-L1 is FDA-approved as a companion diagnostic for NSCLC, bladder cancer, triple-negative breast cancer, cervical cancer, and gastric/GEJ cancer [27].

However, the type of PD-L1-expressing cells to be measured differs depending on the carcinoma. The immunohistochemistry for PD-L1 is based on PD-L1 staining on tumor cells for NSCLC. In contrast, triple-negative breast cancer was approved based on tumor-infiltrating immune cells, and cervical cancer was approved based on a combined tumor and immune cell percentage score [27,28,29].

The problem is the heterogeneity of PD-L1 expression within and between tumors, and PD-L1 expression itself is transient and changes over time and space due to the immune response [30] (Table 2).

## 4. Tumor Mutation Burden

Genetic mutations in tumors can generate immunogenic neoplastic antigens, which have been investigated as predictive biomarkers of the response to ICIs [33]. The KEYNOTE-158 study showed that pembrolizumab was effective in treating tumors of anal, bile duct, cervical, endometrial, mesothelioma, neuroendocrine, salivary, small cell lung, thyroid, and vulvar origins that had high oncogene mutation scores (TMB-high, ≥10 mutations per megabase). Pembrolizumab was also effective in tumors of salivary, small-cell lung, thyroid, and vulvar origin with high oncogene mutation scores (TMB-high) [23]. The FDA approved pembrolizumab monotherapy in 2020 for the treatment of adult and pediatric patients with TMB-high, previously unresectable, or metastatic solid tumors [31,32]. However, TMB-high has not been demonstrated in all cancers; glioma, prostate cancer, and breast cancer were not included in the KEYNOTE-158 study. McGrail et al. divided TMB-high tumors into two groups, those in which the neoantigen levels correlated with the number of CD8 T cells in the tumor and those in which the number of CD8 T cells did not correlate, and analyzed the effect of treatment with ICIs [48]. Some patients with TMB do not respond to PD-L1 antibodies or anti-CTLA4 [49,50]. Their findings suggest that TMB-high does not uniformly predict the efficacy of ICI therapy (Table 2).

## 5. High-Frequency Microsatellite Instability and DNA Mismatch Repair

Microsatellites are repeats of a few nucleotides in DNA. In the case of defective DNA mismatch repair (MMR), the number of microsatellite repeats differs from the normal number of DNA repeats because of the inability to repair mistakes during DNA replication [51]. MSI is usually caused by germline mutations in components of mismatch repair (MSH2, MSH6, MLH1, PMS2) in patients with Lynch syndrome or somatic hypermethylation of the MLH1 promoter [34]. This is called MSI-high [52]. It is more frequent in gastrointestinal and gynecological cancers [53]. MSI-high colorectal cancer (CRC) has shown a higher mutation load than microsatellite stable CRC, and a high somatic mutation load was associated with prolonged progression-free survival (PFS) [54]. A phase III randomized controlled trial (KEYNOTE-177) was conducted in patients with unresectable or metastatic MSI-H/dMMR CRC [24]. Patients with MSI-H/dMMR metastatic CRC who were treated with pembrolizumab as a first-line therapy demonstrated a significantly longer PFS than those treated with chemotherapy. Pembrolizumab has become the first-line treatment for unresectable or metastatic MSI-H/dMMR CRC [24]. Pembrolizumab was also approved by FDA in 2017 for the treatment of patients with unresectable or metastatic, microsatellite instability-high (MSI-H), or mismatch repair deficient (dMMR) solid tumors [25]. MSI-high is a predictive biomarker for response to ICI [33,34].

Dostarlimab, an anti-PD-1 monoclonal antibody, was administered for 6 months as neoadjuvant therapy to patients with deficient mismatch repair (dMMR)–stage II or III rectal adenocarcinoma. After 6 months of treatment, twelve patients showed clinically complete responses [55]. This result suggests that dMMR is a predictive marker for the efficacy of preoperative immunotherapy for rectal cancer (Table 2).

## 6. Intestinal Bacteria

Approximately 100 trillion different bacteria coexist in the human intestine, forming intestinal microflora (also called the intestinal flora) weighing 1.5 to 2 kg [56]. However, with advancements in technology, the number of intestinal bacteria in the body is now being disputed, and this number may be revised in the future. How these intestinal bacteria coexist with humans is not known. However, it is an extremely important relationship, and it has become known in recent years that the disruption of the relationship with the intestinal microflora causes inflammatory bowel disease, rheumatic diseases, obesity, diabetes, atopy, and allergies. This disordered state of the intestinal microflora is called dysbiosis, which means a breakdown in the composition of the intestinal bacteria [57].

Rapid progress in the analysis of gut microbiota began with the advent of next-generation sequencing. In other words, it came about as a result of the instantaneous availability of large amounts of genetic analysis techniques. Before the full-scale introduction of next-generation sequencing, it was known that the bacterial genome, which consists of several million base pairs, contains a polymorphic region of approximately 1600 base pairs called the 16S ribosomal RNA region. The 16S ribosomal RNA region has nine hypervariable regions consisting of tens to hundreds of base pairs, which have characteristic sequences depending on the type of bacteria. The sequences of these hypervariable regions are conserved among the same bacterial species, and it is believed that the bacterial species can be identified by reading and analyzing the full or partial length of the 16S region without sequencing the entire bacterial genome.

**Figure 1:**
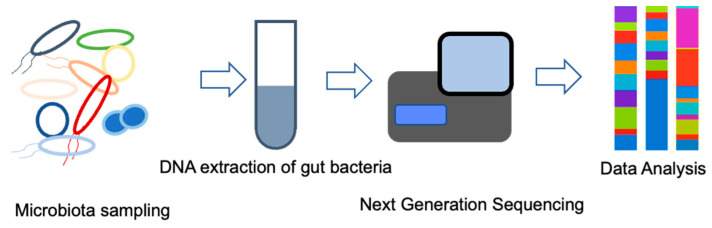
Microbiome identification by 16S rRNA gene analysis.

In the analysis of intestinal microflora by 16S metagenomics, the DNA of enterobacteria in feces is extracted and purified as a template, and the gene is amplified using primers designed to amplify a part of the 16S region. The gene sequences contained in the library are read using a next-generation sequencer and matched with a database to identify the type of bacteria. Using this genetic testing method, it is possible to detect DNA fragments of living bacteria in feces and DNA fragments of dead bacteria that cannot be detected by the culture method, and it is believed to provide accurate information regarding the intestinal microflora. The process of gene extraction and purification from feces, gene amplification, purification and quantification of amplified products, library preparation, sequencing, identification, and calculation of the percentage of bacteria using analysis software can be completed in about 3 days. In the future, it is expected that the information obtained will be successively compiled into databases (Figure 1). Recent technological innovations have led to the accumulation of a considerable amount of information on intestinal bacteria and diseases, especially cancer [58,59].

## 7. Composition of the Intestinal Microflora

The formation of human gut microbiota begins immediately after birth. The gut flora formed in the neonatal period is not constant throughout life, and the composition of the constituent bacteria changes with age [58]. The formation process of the intestinal flora can vary and is affected by various environmental factors such as the duration of fetal life, mode of delivery, and mode of lactation [59]. According to the research on dysbiosis described above, it is important to have a good balance between the so-called good and bad bacteria in the intestinal microbiota, and it has often been reported that oligotrophic anaerobic bacteria (fermentative bacteria) are good bacteria, whereas commensal anaerobic bacteria are bad bacteria. However, with recent advances in the study of dysbiosis, either this rule does not necessarily apply, or the terms “good” and “bad” bacteria themselves are being less commonly used [36,37,38,60,61,62,63].

The effect of gut bacteria on ICI therapy has been reported by research groups in the USA and in France [35,36,37,38]. They have claimed that certain gut bacteria may modulate the clinical effects of anti-PD-1 antibodies. However, the gut microbiota reported by each group was different and has not been identified yet. In addition, the pattern of gut microbiota differs among countries and diets (Table 2).

## 8. Microbiota Is a Potential Biomarker of ICIs

Various studies have been conducted to explore how intestinal bacteria act on the immune system. It has been shown that the involvement of single-chain fatty acids is a major mechanism of action. It is believed that the actions of single-chain fatty acids may change due to differences in their receptors, but the details need to be clarified in future studies [64].

It is important to note that many researchers, including us, are currently conducting research aimed at identifying the immune states that are likely to cause immune-related adverse events (irAEs) and those that are likely to result in effective immunotherapy by analyzing the intestinal bacteria. Research is underway to induce a state in which irAEs are less likely to occur, and immunotherapy is more likely to be effective by using various therapies, including the modification of the intestinal microbiota. If the optimal immune conditions can be estimated using biomarkers, treatment strategies, especially the management of adverse events, will become easier.

## 9. Attenuation of ICI Efficacy Via the Effects of Antibiotics on Gut Bacteria

In the field of cancer research, data are emerging that gut microbiota may be highly correlated with the therapeutic effects of cancer immunotherapy [65,66,67]. It is also believed that intestinal bacteria may be involved in cancers of the esophagus, stomach, and many other organs [68].

Interestingly, a growing body of evidence suggests that antibiotics have a strongly negative impact on gut bacteria [69]. In addition, there are some interesting data on the negative effects of antibiotics during treatment with ICIs.

We compared NSCLC patients receiving anti-PD-1 antibody treatment with and without antibiotics and found that both the PFS and overall survival (OS) were significantly lower in the group that used some antibiotics within 3 weeks before and after the start of anti-PD-1 antibody treatment than those in the group that did not use antibiotics. In other words, antibiotic use hampers the efficacy of ICIs [70]. Using antibiotics is known to cause dysbiosis of the gut microbiota [71], suggesting that dysbiosis could reduce the efficacy of PD-1 antibody therapy.

## 10. Metabolites of Gut Bacteria Affect Immunity

**Figure 2:**
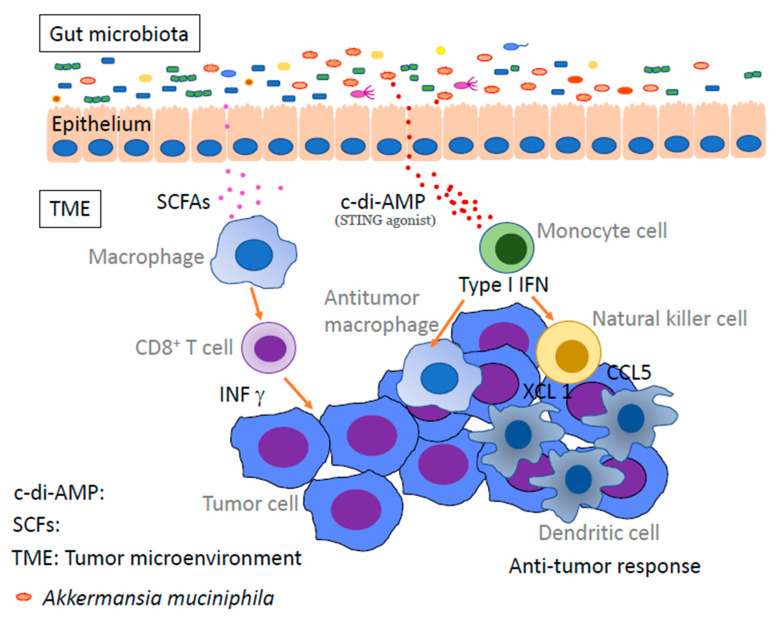
Gut microbiota enhances antitumor immunity via microbial metabolites. Microbe-derived short-chain fatty acids and STING agonists enhance anti-tumor immunity by activating monocytes, dendritic cells, anti-tumor macrophages, and CD8 T cells in the tumor microenvironment. TME: tumor microenvironment, SCFAs: short-chain fatty acids, c-di-AMP: c-di-adenosine monophosphate.

The above-mentioned ectopic anaerobic bacteria produce short-chain fatty acids (SCFAs) by fermentation using dietary fiber as a nutrient source [72,73,74,75]. These SCFAs include propionate, acetate, and butyrate, all of which have fewer than six carbons and are believed to influence immune activity and regulation. Certain microbiota, such as *Akkermansia muciniphila*, modulate the activation of monocytes, dendritic cells, and macrophages in the cancer microenvironment via bacterially derived substances. Thereby, anti-tumor immunity is activated, and the efficacy of anti-PD-1 immunotherapy is enhanced [76] (Figure 2). In any case, SCFAs play important roles in human immunity and homeostasis, such as the induction of Tregs and type 1 helper T cells and the maintenance of intestinal epithelial cell proliferation [64]. However, SCFAs have not been shown to be effective against tumors, and there are still many unresolved questions on the relationship between dietary fiber and anti-tumor effects. SCFAs certainly play a vital role, and research on the importance of fiber in the diet and the effects of each SCFA on immunity is becoming increasingly important [77,78]. Recently, in addition to SCFAs, metabolites produced by intestinal bacteria have been extensively studied. It is known that commensal anaerobes have a low expression of digestive enzymes that digest dietary fiber [79]. It is also known that they utilize nutrient sources that are abundant in Westernized diets, such as monosaccharides, disaccharides, fats, proteins, and alcohol, rather than dietary fiber.

These SCFAs are generally believed to increase anti-tumor activity, but there are also data that they may inhibit some types of cancer immunity or in certain conditions. A representative example of this is a mouse study that demonstrated that sodium butyrate inhibited anti-CTLA-4-induced dendritic cell (DC) maturation and T-cell priming [40]. Therefore, further studies are needed to elucidate the mechanism underlying the effects of individual SCFAs on cancer immunity.

## 11. Other Potential Biomarkers for ICIs

The analysis of immune cells in peripheral blood is a non-invasive method of predicting the outcome of immunotherapy treatments [80]. In patients with stage IV melanoma, the high frequency of CD14^+^ CD16^−^ HLA-DR^hi^ monocytes in peripheral blood samples before anti-PD-1 immunotherapy was a strong predictor of PFS and OS in response to PD-1 immunotherapy [44]. In patients with metastatic melanoma, a higher fraction of activated CD4 effector memory T cells, which lack expression of CCR7 and SELL (CD62L), is associated with a higher incidence of severe irAEs within 3 months [81]. In patients with NSCLC and gastric cancer, significance was found to correlate with decreased soluble PD-L1 levels and tumor regression after four cycles of PD-1 Ab [41]. Changes in plasma soluble PD-1 concentrations before and after two and four cycles of anti-PD-1 antibody treatment were significantly correlated with tumor size progression [42]. PD-L1^+^ CD14^+^ monocytes in peripheral blood are correlated with shorter survival in patients treated with anti-PD-1 antibodies, including NSCLC, gastric cancer, melanoma, parotid cancer, and bladder cancer [43]. The neutrophil-to-lymphocyte ratio (NLR) has the potential to predict treatment outcomes in patients receiving ICIs [45,46,82]. Furthermore, the probability of obtaining an ICI benefit is significantly higher in the NLR low/TMB high group compared to the NLR high/TMB low group (OR = 3.22; 95% CI, 2.26–4.58; *p* < 0.001) [47]. CXCL13 in the tumor microenvironment of many different types of cancer, and CXCL13 is associated with favorable outcomes in cancer patients treated with ICIs [39].

## 12. Conclusions

Although PD-L1, TMB, and MSI-high are predictive markers for the efficacy of ICIs, they are not common in many carcinomas. Recently, it has been recognized that the gut microbiota functions as a single organ. It is becoming clear that the gut microbiota influences many diseases, including cancer, through its metabolism. Metabolites of the gut microbiota have been shown to modulate immunity and have a strong effect on the therapeutic efficacy of ICIs. Accumulating evidence suggests that the gut microbiota may act as a novel predictive marker for the efficacy of ICIs.

## Figures and Tables

**Table 1:** Types of cancer effective for ICI (monotherapy).

Drug	Cancer Type	Reference
Nivolumab (Anti-PD-1)	Melanoma	[8]
	NSCLC	[6]
	Hodgkin lymphoma	[10]
	Renal cell carcinoma	[12]
	Gastric cancer	[11]
Pembrolizumab (Anti-PD-1)	NSCLC	[7]
	Head and neck squamous cell carcinoma	[14]
	Urothelial carcinoma	[13]
	TMB-High	[23]
	MSI-high/dMMR	[24,25]
Atezolizumab (Anti-PD-L1)	NSCLC	[15,17]
Avelumab (Anti-PD-L1)	Merkel cell carcinoma	[16]
Ipilimumab (Anti-CTLA-4)	Melanoma	[21]

**Table 2:** Predictive biomarkers for treatment of immune checkpoint inhibitors.

Biomarker	Drug	Cancer type	Predictive	Poor predictive	References
**Tumor biomarkers**					
PD-L1 Expression on pre-treatment tumor cells	PD-1/PD-L1 Ab	Melanoma, NSCLC, RCC	Increased		[8,26,27]
PD-L1 Expression on tumor-infiltrating immune cells		Triple-negative breast cancer	Increased		[28]
PD-L1 Expression on a combined tumor and immune cell percentage score		Cervical cancer	Increased		[29]
TMB-high	PD-1 Ab	TMB-high solid tumors	Increased		[31,32]
MSI-H/dMMR	PD-1 Ab	MSI-H/dMMR solid tumors	Increased		[33,34]
**Gut microbiota**					
*Faecalibacterium* spp.		Melanoma	Increased		[35]
*Akkermansia muciniphila* and *Enterococcus hirae*	PD-1 Ab	NSCLC, Urothelial carcinomas	Increased		[36]
*Ruminococcaceae family*	PD-1 Ab	Melanoma	Increased		[37]
*Bifidobacterium longum, Collinsella aerofaciens, Enterococcus faecium*	PD-1 Ab	Melanoma	Increased		[38]
Gut Microbiota Diversity	PD-1 Ab	Melanoma	Increased		[39]
**Microbiota metabolite**					
Elevated serum levels of propionate and butyrate	CTLA-4 Ab	Melanoma		Increased	[40]
**Immune biomarkers**					
Plasma Levels of Soluble PD-L1 at four cycles of treatment	PD-1 Ab	NSCLC, Gastric cancer, Bladder cancer	Decreased		[41]
Plasma Soluble PD-1	PD-1 Ab	NSCLC, Gastric cancer, Bladder cancer		Increased	[42]
PD-L1^+^ CD14^+^ monocytes	PD-1 Ab	NSCLC, Gastric cancer, Melanoma, Parotid cancer, Bladder cancer		Increased	[43]
CD14^+^ CD16^−^ HLA-DR^hi^ monocytes	PD-1 Ab	Melanoma	Increased		[44]
The neutrophil-to-lymphocyte ratio (NLR)	CTLA-4 Ab/PD-1 Ab	Melanoma		Increased	[45,46]
	PD-1 Ab	NSCLC, RCC, Sarcoma, Bladder, Colorectal		Increased	[47]
CXC chemokine ligand 13	PD-1/PD-L1 Ab, CTLA-4 Ab	Bladder cancer, Urothelial carcinoma, Melanoma, Head and neck cancer, NSCLC, RCC, Colorectal cancer, Breast cancer	Increased		[39]

Abbreviations: CI, NSCLC, non-small cell lung cancer; RCC, Renal cell carcinoma; TMB, Tumor mutation burden; MismatchRepairDeficien:dMMR; Microsatellite instability-high, MSI-H; CXCL, CXC chemokine ligand; PD-1, programmed cell death protein 1; CTLA4, cytotoxic T lymphocyte-associated protein.

## Data Availability

Not applicable.
